# CUP-SHAPED COTYLEDON1 (CUC1) and CUC2 regulate cytokinin homeostasis to determine ovule number in Arabidopsis

**DOI:** 10.1093/jxb/ery281

**Published:** 2018-07-26

**Authors:** Mara Cucinotta, Silvia Manrique, Candela Cuesta, Eva Benkova, Ondrej Novak, Lucia Colombo

**Affiliations:** 1 Dipartimento di Bioscienze, Università degli Studi di Milano, Milano, Italy; 2 Institute of Science and Technology Austria (IST Austria), Klosterneuburg, Austria; 3 Laboratory of Growth Regulators, Centre of the Region Haná for Biotechnological and Agricultural Research, Faculty of Science of Palacký University and Institute of Experimental Botany of the Czech Academy of Sciences, Academy of Sciences of the Czech Republic, Šlechtitelů, Olomouc, Czech Republic

**Keywords:** Arabidopsis, cytokinin, hormones, ovule development, pistil development, transcriptional regulation

## Abstract

Seeds derive from ovules upon fertilization and therefore the total number of ovules determines the final seed yield, a fundamental trait in crop plants. Among the factors that co-ordinate the process of ovule formation, the transcription factors CUP-SHAPED COTYLEDON 1 (CUC1) and CUC2 and the hormone cytokinin (CK) have a particularly prominent role. Indeed, the absence of both CUC1 and CUC2 causes a severe reduction in ovule number, a phenotype that can be rescued by CK treatment. In this study, we combined CK quantification with an integrative genome-wide target identification approach to select Arabidopsis genes regulated by CUCs that are also involved in CK metabolism. We focused our attention on the functional characterization of *UDP-GLUCOSYL TRANSFERASE 85A3* (*UGT85A3*) and *UGT73C1*, which are up-regulated in the absence of CUC1 and CUC2 and encode enzymes able to catalyse CK inactivation by *O*-glucosylation. Our results demonstrate a role for these *UGT*s as a link between CUCs and CK homeostasis, and highlight the importance of CUCs and CKs in the determination of seed yield.

## Teaser

CUC1 and CUC2 regulate ovule and seed number by modulating the expression of genes involved in the balance between active and inactive forms of glycosylated cytokinin.

## Introduction

Ovule primordia emerge as lateral organs from meristematic placental tissue following a series of periclinal cell divisions (Schneitz *et al.*, 1995). The placenta, in turn, is derived from the carpel margin meristem (CMM), whose formation is known to be controlled by the interaction of genetic and hormonal networks (reviewed in Reyes-Olalde *et al.*, 2013). In recent decades, the fundamental role of hormones such as cytokinins (CKs), auxins, and brassinosteroids in determination of ovule number has been studied, and several transcription factors modulating the hormonal responses have been identified (reviewed in Cucinotta *et al.*, 2014).

The transcription factors CUP SHAPED COTYLEDON1 (CUC1) and CUC2 have been found to be involved in the establishment of boundaries between organs. Indeed, the characteristic phenotype of *cuc1cuc2* double-mutants is the presence of fused cotyledons (Aida *et al.*, 1997). During ovule development, they form part of a network that regulates ovule primordia initiation (Galbiati *et al.*, 2013). *CUC1* and *CUC2* are broadly expressed in the placental tissue but, after primordia formation, their expression is limited to the boundaries between ovules (Nahar *et al.*, 2012; Galbiati *et al.*, 2013). Pistils in which *CUC2* is mutated and *CUC1* is knocked down with an RNAi construct under the control of the ovule/placenta-specific SEEDSTICK promoter (*cuc2 pSTK::RNAi-CUC1*) have a reduced number of ovules relative to the wild-type (Galbiati *et al.*, 2013). In *cuc2 pSTK::RNAi-CUC1* the pistil size is not affected but the space between adjacent primordia increases, leading to a reduced ovule density.

In addition, CUCs promote expression of the auxin efflux carrier *PIN1*, which is necessary for auxin distribution in the placenta (Galbiati *et al.*, 2013). *PIN1* expression in the pistil is also activated by CKs through CYTOKININ RESPONSE FACTOR 2 (CRF2) and CRF6 (Bencivenga *et al.*, 2012; Cucinotta *et al.*, 2016), and CK treatment rescues the *cuc2 pSTK::RNAi-CUC1* ovule-number phenotype by restoring *PIN1* expression (Galbiati *et al.*, 2013), suggesting that CUCs could regulate CK abundance. Several studies have clearly demonstrated that CKs are positive regulators of ovule number (Bartrina *et al.*, 2011; Bencivenga *et al.*, 2012; Galbiati *et al.*, 2013). Mutants impaired in CK signalling and response have a drastically reduced number of ovules (Riefler *et al.*, 2006; Kinoshita-Tsujimura and Kakimoto, 2011; Bencivenga *et al.*, 2012; Cucinotta *et al.*, 2016). In contrast, plants with higher CK content form more ovules. For instance, the total seed yield of a double-mutant for two CK degradation enzymes, the cytokinin oxidase/dehydrogenases CKX3 (encoded by *AT5G56780*) and CKX5 (encoded by *AT1G75450*), is twice that of the wild-type (Bartrina *et al.*, 2011).

Here, we report that mutation of *CUC1* and *CUC2* alters CK homeostasis in Arabidopsis. We quantified the concentration of CKs in the double-mutant and performed an RNA-seq experiment to identify deregulated genes involved in CK homeostasis that have an effect on ovule number determination. In particular, we focused our attention on the role of two members of the *UDP-GLUCOSYL TRANSFERASE* family (*UGT85A3* and *UGT73C1*) that catalyse the reversible inactivation of zeatin-type CKs by *O*-glucosylation (Hou *et al.*, 2004), showing that both factors are involved in ovule number determination.

## Materials and methods

### Plant material and growth conditions

*Arabidopsis thaliana* ecotype Columbia-0 (Col-0, wild-type) and transgenic lines were growth in soil at 22 °C under long-day conditions (16/8 h light/dark). *cuc2 pSTK::RNAi-CUC1* has been described previously (Galbiati *et al.*, 2013). The mutant line SAIL_241_G09 renamed here as *ugt85a3-1* carries a T-DNA insertion in the first exon of the *UGT85A3* gene (AT1G22380); SALK_070258, renamed here as *ugt85a3-2*, carries a T-DNA insertion in the second exon of *UGT85A3* that generates rearrangement of the gene and of the T-DNA itself (Supplementary Fig. S2 at *JXB* online). Mutant *ugt73c1* corresponds to SALK_207721C and carries an insertion at the beginning of the coding sequence of *UGT73C1* (AT2G36750), which is an intron-less gene (Supplementary Fig. S2). Seeds of *ugt85a3-1*, *ugt85a3-2*, and *ugt73c1* were obtained from NASC (http://arabidopsis.info/) and a list of primers used for genotyping is available in Supplementary Table S5. To construct *35S::UGT85A3* and *35S::UGT73C1*, a genomic fragment was amplified with the primers AtP_4903/AtP_4904 and AtP_4907/AtP_4908 and cloned into the Gateway® destination vector pB2GW7. All constructs were verified by sequencing and used to transform wild-type plants using the floral dip method (Clough and Bent, 1998).

### Protoplast transfection

Protoplast preparation and transient expression experiments were performed as described by Galbiati *et al.* (2013).

### RNA extraction, cDNA library preparation, and sequencing for RNA-seq

Total RNA was extracted from three biological replicates (0.5 g) from both wild-type and *cuc2 pSTK::RNAi-CUC1* mutant pre-fertilization pistils, using a NucleoSpin RNA Plant kit (MachereyNagel) according to the manufacturer’s instructions. RNA integrity was analysed by gel electrophoresis and spectrophotometrically before submission to the sequencing facility. Sequencing libraries were prepared with a TruSeq RNA Sample Prep kit (Illumina Inc.) and subsequently sequenced on an Illumina HiSeq2000 (50 bp single-read) by IGATech (http://www.igatechnology.com/).

Mapping of short reads, quality analysis, and assessment of gene expression were performed as described by Mizzotti *et al.* (2014). Evaluation and treatment of raw data was performed using CLC Genomics Workbench v.4.7.1 (https://www.qiagenbioinformatics.com/products). After filtering, high-quality reads were mapped onto the Arabidopsis genome (TAIR10). Approximately 20 million reads of each sample that mapped with ≤2 mismatches were used for further analyses. The fold-change and differential expression values between the wild-type and the *cuc2 pSTK::RNAi-CUC1* mutant were calculated in terms of the reads per kilobase of million mapped reads (RPKM) of the corresponding transcripts. To obtain statistical confirmation of the differences in gene expression, *P*-values were computed. We applied a threshold value of *P*=0.05 to ensure that differential gene expression was maintained at a significant level for the individual statistical tests. Transcripts that exhibited an estimated Absolute Fold-Change ≥1.5 (i.e. 2 mapped reads per kilobase of mRNA) were considered to be significantly differentially expressed.

### Expression analysis

Total RNA was extracted from pistils or inflorescences at pre-fertilization stages using a NucleoSpin RNA Plant kit (Macherey-Nagel) and equal amounts of each sample were reverse-transcribed using the GoScript™ Reverse Transcription System (Promega). The cDNAs were standardized relative to *ACTIN2-8* (*ACT2-8*) and *UBIQUITIN10* (*UBI10*) transcripts, and gene expression analysis was performed using a Bio-Rad iQ5 Multicolor real-time PCR detection system with GeneSpin SYBR Green PCR Master Mix. RT-PCR primers are listed in Supplementary Table S5.

### Cytokinin quantification

Quantification of CK metabolites was performed as described by Svačinová *et al.* (2012), including the modifications of Antoniadi *et al.* (2015). Fresh plant tissue (20 mg) was homogenized and extracted in 1 ml of 80% methanol together with a cocktail of stable isotope-labelled internal standards (0.25 pmol of CK bases, ribosides, N-glucosides, and 0.5 pmol of CK O-glucosides, nucleotides added per sample). The extracts were purified using an Oasis MCX column (30 mg 1 ml^–1^; Waters, Milford, MA, USA) conditioned with 1 ml each of 100% methanol and H_2_O, equilibrated sequentially with 1 ml of 50% (v/v) nitric acid, 1 ml of H_2_O, and 1 ml of 1M HCOOH. After sample application onto an MCX column, unretained compounds were removed by a wash step with 1 ml of 1M HCOOH and 1 ml 100% MeOH. Pre-concentrated analytes were eluted by two-step elution using 1 ml of aqueous 0.35 M NH_4_OH and 2 ml of 0.35M NH_4_OH in 60% (v/v) MeOH solution. The eluates were then evaporated to dryness *in vacuo* and stored at –20 °C. Separation was performed on an Acquity UPLC^®^ System (Waters) equipped with an Acquity UPLC BEH C18 column (150 × 2.1 mm, 1.7 μm; Waters), and the effluent was introduced into the electrospray ion source of a triple quadrupole mass spectrometer Xevo™ TQ-S MS (Waters). Analytes were quantified using diagnostic MRM transitions of precursor and appropriate product ions using stable isotope-labelled internal standards for reference (Novák *et al.*, 2008). Four independent biological replicates were performed.

## Results

### CUC1 and CUC2 induce a CK response *in vivo*

CUCs promote expression of the auxin efflux carrier *PIN1*, which is necessary for auxin distribution in the placenta, and when both *CUC1* and *CUC2* are silenced in the placenta the number of ovules formed is significantly reduced (Galbiati *et al.*, 2013). CK treatment (benzyl adenine, BAP) restores ovule number in *cuc2 pSTK::RNAi-CUC1* plants by re-establishing the correct level of *PIN1* expression (Galbiati *et al.*, 2013; Cucinotta *et al.*, 2016). BAP treatment does not have any effect if *PIN1* is mutated (Bencivenga *et al.*, 2012; Cucinotta *et al.*, 2016), as is the case in *pin1-5* plants. One possible explanation is that CKs act downstream of CUC1 and CUC2 to induce *PIN1* expression.

**Fig. 1.**
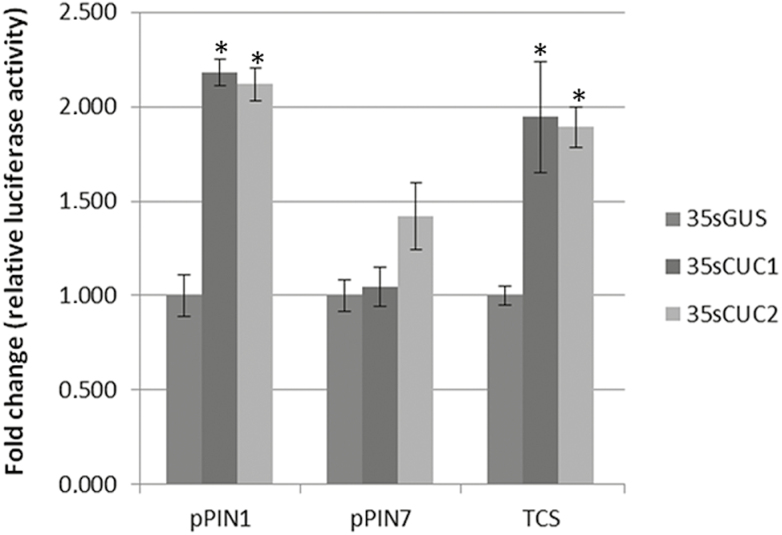
*CUC* overexpression enhances cytokinin response. Luciferase activity in tobacco BY-2 protoplasts transiently transformed with *pPIN1::LUC*, *pPIN7::LUC*, or *pTCS::LUC* expressing *p35s::GUS*, *p35s::CUC1*, or *p35s::CUC2*. *TCS* activity, as well as *PIN1*, is enhanced by *CUC1* and *CUC2* overexpression compared to the *p35s::GUS* control, while *PIN7* expression remains at basal levels. The fold-change is normalized to the activity of luciferase (LUC) in *p35s::GUS* protoplasts. Data are means (±SE), *n*=8 separate transfection events and measurements. Significant differences between mock and effector constructs (35s::*CUC1*, 35s::*CUC2*) were determined using Student’s *t*-test (*P<0.01).

To elucidate whether CUC1 and CUC2 are able to induce a CK response *in vivo*, we performed transient expression assays in BY-2 tobacco (*Nicotiana benthamiana*) protoplasts. We used the ‘two-component system (TCS) signalling sensor’ promoter fused to the luciferase (*LUC*) gene as a CK-response reporter (Müller and Sheen, 2008). The TCS is a synthetic promoter that reflects the transcriptional activity of type-B response regulators, the final targets of the CK signalling cascade. Protoplasts were transformed either with a *35S::CUC1/2* construct or a *35S::GUS* construct as a negative control. Protoplasts overexpressing *CUC1* or *CUC2* showed a significant increase of *TCS::LUC* expression compared to controls (Fig. 1). We used the induction of *pPIN1::LUC* by both CUC1 and CUC2 as a positive control and *pPIN7::LUC* as negative control (Galbiati *et al.*, 2013). The results of the protoplast assay suggested that CUC1 and CUC2 were positive regulators of CK response.

### CUC1 and CUC2 influence CK homeostasis

**Fig. 2.**
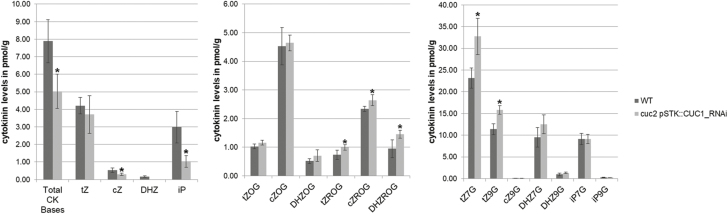
Cytokinin content of pre-fertilization inflorescences of wild-type (WT) Col-0 and the *cuc2 pSTK::RNAi-CUC1* mutant. Total cytokinin (CK) levels and the levels of relevant CK metabolites are shown: (A) active CK bases, (B) CK *O*-glucosides, and (C) CK *N*-glucosides. Data are means (±SD) of four biological replicates. Significant differences between WT and the *cuc2 pSTK::RNAi-CUC1* mutant were determined using Student’s *t*-test (*P<0.01).

The increased expression of the *TCS::LUC* reporter in protoplasts overexpressing *CUC1* and *CUC2* could be explained by the action of the two transcription factors on metabolism of, signalling by, or the response to CK. To investigate whether CUC1 and CUC2 modulated CK homeostasis, we quantified CK levels with LC-MS. Pre-fertilization inflorescences of *cuc2 pSTK::RNAi-CUC1* plants were compared to those of the wild-type. *cuc2 pSTK::RNAi-CUC1* plants showed a significant reduction in total active CK bases, particularly *cis*-zeatin (*c*Z) and isopentenyladenine (iP) relative to the wild-type (Fig. 2A). In addition, in the *cuc2 pSTK::RNAi-CUC1* background a consistent increase in the level of three types of *O*-glucosylated CK ribosides (*trans*-zeatin ROG, *cis*-zeatin ROG, and dihydrozeatin ROG) was detected (Fig. 2B). *O*-glucosylation is a reversible reaction that occurs at the hydroxyl group on the side-chains of zeatin or dihydrozeatin. CK *O*-glucosides (*t*ZROG, *c*ZROG, and DHZROG) are inactive in bioassays and play a role in maintaining CK homeostasis (Mok and Mok, 2001; Spíchal *et al.*, 2004; Šmehilová *et al.*, 2016). Moreover, in *cuc2 pSTK::RNAi-CUC1* plants the content of inactive *N*-glucosylated *trans*-zeatin (tZ7G and tZ9G) was also higher than wild-type levels, together with a significant increase in total CK *N*-glucosides (Supplementary Table S1). *N*-glucosylation of cytokinin occurs on the nitrogen at positions *N*7 or *N*9 of the purine ring and, unlike *O*-glucosylation, is thought to be irreversible. The level of the other CK metabolites did not change between the wild-type and mutant (Supplementary Table S1). Taken together, the results of CK quantification indicated that in *cuc2 pSTK::RNAi-CUC1* plants there was an increase in the amount of inactive CK glucosides at the expense of active CK bases, suggesting that CUCs might be involved in the regulation of CK homeostasis.

### Identification of CUC1/2 downstream targets related to CK metabolism

To gain insight on how CUCs regulate CK homeostasis at the genetic level, we performed a transcriptomic analysis by RNA-seq. The transcriptome of wild-type pistils was compared to those from *cuc2 pSTK::RNAi-CUC1* plants in order to identify genes downstream of CUC1 and CUC2 linked to CK metabolic pathways during pistil and ovule development.

In order to enrich the dataset in genes involved in ovule development, unfertilized pistils from developmental stages 8 to 12 (according to Roeder and Yanofsky, 2006) were dissected and utilized for RNA extraction.

Bioinformatic analysis revealed varying levels of silencing of *CUC1* in the biological replicates, probably due to the fact that we used RNAi, which typically leads to variable levels of silencing that tend to decrease over time. Nonetheless, the analysis (see Methods for details) identified 416 differentially expressed genes (DEGs) in the *cuc2 pSTK::RNAi-CUC1* mutant with a fold-change higher than 1.5 and a *P*-value of 0.05 or less. Of these, 251 genes were significantly up-regulated (Supplementary Table S2), whereas 165 were found to be significantly down-regulated (Supplementary Table S3).

There are few known direct targets of CUCs during pistil and ovule development that could be used to validate the dataset. However, *LATERAL SUPPRESSOR* (*LAS*) is known to be a direct target of CUCs in the initiation of axillary meristems (Raman *et al.*, 2008), and this was indeed down-regulated almost 5-fold (–4.723) in our dataset, supporting the validity of the data.

**Fig. 3.**
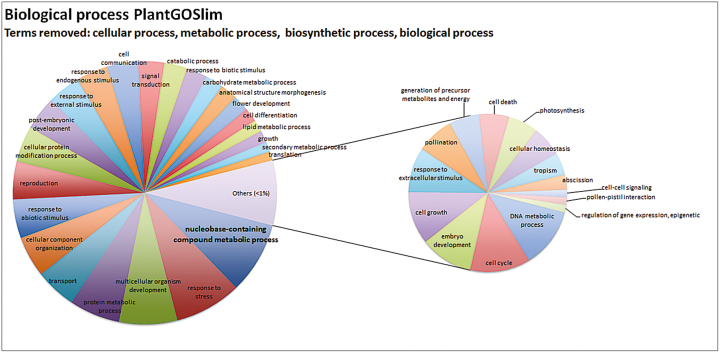
Relative abundance of gene ontology (GO) terms belonging to the ‘biological process’ category associated with differentially expressed genes in the *cuc2 pSTK::RNAi-CUC1* mutant compared with the Col-0 wild-type. Functional categorization of GO terms was performed using the AmiGO tool and the PlantGOSlim vocabulary. Generic terms such as ‘biological process’ and ‘cellular process’ have been removed for clarity. Categories with an abundance lower than 1% (‘Others’) are plotted separately for greater clarity.

To gain more insight into the data and the potential functions of genes downstream of CUC1 and CUC2, a functional categorization of the gene ontology (GO) terms (Ashburner *et al.*, 2000; The Gene Ontology Consortium, 2017) associated with the DEGs was performed using version 1.8 of the AmiGO tool (Carbon *et al.*, 2009) and the PlantGoSlim vocabulary (Supplementary Table S4). The categorization of the ‘biological process’ terms is shown in Fig. 3. Generic terms such as ‘biological process’ (GO:0008150) and ‘cellular process’ (GO:0009987) have been removed from the graph to highlight other more relevant terms (the complete list of terms is shown in Supplementary Table 4). The categories observed fitted with the tissues sampled and with previously described roles of CUC1 and CUC2 in these processes. For example, categories such as ‘reproduction’ (GO:0000003) and ‘flower development’ (GO:0009908) were abundantly represented. Categories related to hormonal responses such as ‘response to endogenous stimulus’ (GO:0009719) and ‘signal transduction’ (GO:0007165) were also represented, but terms directly related to the response to specific hormones were absent.

After removing generic terms, ‘nucleobase-containing compound metabolic process’ (GO:0006139) was the most abundant. This term is defined as any cellular metabolic process involving nucleobases, nucleosides, nucleotides, and nucleic acids. This includes metabolic precursors or derivates of cytokinins, in agreement with the possible role of CUCs in control of CK homeostasis as revealed by CK quantification.

Overall, the categorization of the terms associated with the DEGs fitted with the role of CUCs and the tissue sampled, and revealed alterations in genes related with CK metabolism. None of these terms were found to be statistically over-represented in an enrichment analysis performed with the PANTHER 13.0 software against the whole Arabidopsis genome (Mi *et al.*, 2017). This was probably because we could only identify a moderate number of DEGs due to the intrinsic variability of the samples lowering the statistical power of the analysis. Nonetheless, these data provide insight and directions for future studies.

Combining the information from the RNA-seq and CK quantification, we searched for DEGs that might be related to CK homeostasis pathways for further study. From the list of up-regulated genes, we selected *UDP-GLUCOSYL TRANSFERASE 85A3* (*UGT85A3*) because it is the closest homolog to UGT85A1 that has been biochemically characterized as a CK-specific UGT that is able to catalyse *O*-glucosylation of CKs (Hou *et al.*, 2004). Another protein with similar biochemical properties is UGT73C1. *UGT73C1* was also up-regulated in our dataset (fold-change +2.904), although initially it was excluded from the list of DEGs due to a *P*-value higher than 0.05 (0.064) (Supplementary Table S2). Given the sample variability and the possible functional relevance of *UGT73C1*, we re-evaluated its expression by qRT-PCR. The expression analysis of *UGT85A3* and *UGT73C1* in the mutant and in the wild-type indeed confirmed the up-regulation of both genes in the *cuc2 pSTK::RNAi-CUC1* plants in the three different biological replicates (Supplementary Fig. S1).

**Fig. 4.**
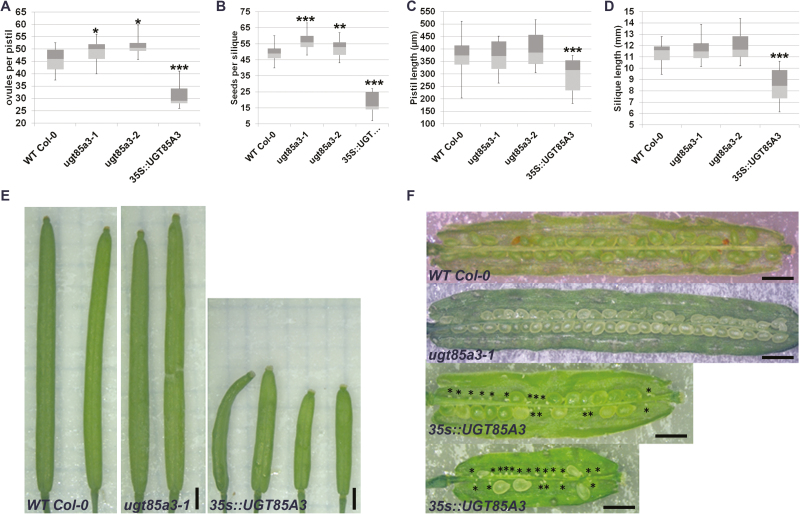
Variation in *UGT85A3* levels affect ovule number and final seed set. (A) Ovule number, (B) seed number, (C) pistil length, and (D) silique length in wild-type (WT) Col-0, *ugt83a3-1*, *ugt85a3-2*, and *35S::UGT85A3*. The box-plots show the first (light grey) and third (dark grey) quartiles separated by the median. Error bars show the maximum and minimum values; (*n*=20 pistils/siliques). Significant differences compared with WT were determined using Student’s *t*-test (*P<0.05, **P<0.01, ***P<0.001). (E) Stereomicroscope images of wild-type, *ugt85a3*, and *35S::UGT85A3* mature siliques. (F) Wild-type and *ugt85a3-1* siliques showing full seed-set, whereas siliques of two representative *35S::UGT85A3* plants display many unfertilized ovules (indicated by asterisks). Scale bars are 1 mm.

In Arabidopsis, the *UGT85A* family consists of six members, of which *A1*, *A2*, *A3*, *A5*, and *A7* are clustered near each other on Chromosome 1 (Woo *et al.*, 2007). The *UGT73C* family is comprised of seven genes, six of which are clustered in tandem on Chromosome 2 (*UGT73C1*–*6*) (Husar *et al.*, 2011). The *O*-glucosyltransferase activity of UGT73C1 and UGT85A1, a close homolog of UGT85A3, have been proven *in vitro*, and they are able to recognize *t*Z, *c*Z, and DHZ, and to form *O*-glucosides (Hou *et al.*, 2004). The high protein-sequence similarity between UGT85A1 and A3 suggests that they evolved by gene duplication and may therefore have similar enzymatic properties (Li *et al.*, 2001). To investigate their role in the determination of ovule number, we analysed two independent *ugt85a3* mutant alleles, the *ugt73c1* mutant (Supplementary Fig. S2) and plants in which *UGT85A3* and *UGT73C1* were overexpressed with the CaMV35S promoter. Phenotypic analysis showed a moderate but significant increase in ovule and seed number in both the *ugt85a3-1* and *ugt85a3-2* independent *ugt85a3* mutant lines (Fig. 4A, B). In particular, *ugt85a3-1* has an increase of 12.5% and 13% in ovules and seeds, respectively, compared to the wild-type, while *ugt85a3-2* developed 7% more ovules and 10% more seeds than the wild-type. No significant differences were detected in pistil and silique lengths in the *ugt85a3* mutants relative to the wild-type (Fig. 4C, D). These results suggested that UGT85A3 may have a role in determining the density of ovules formed inside the pistil, as already demonstrated for CUC1 and CUC2 (Galbiati *et al.*, 2013). The production of more ovules was not accompanied by a greater length of the pistil and did not affect fertility in the *ugt85a3* mutants, which produced a full seed-set and no ovule or seed abortions (Fig. 4D, E). On the contrary, when *UGT85A3* was overexpressed, plant fertility was impaired. Indeed, plants of four independent lines, with high levels of *UGT85A3* expression (Supplementary Fig. S2), showed strong CK deficiency-related pleiotropic phenotypes, such as smaller stature than the wild-type and significant reductions in ovule number and pistil length (Fig. 4A, C). Moreover, in *35S::UGT85A3* the silique length and the seed number were even more reduced due to a large number of unfertilized ovules (Fig. 4B, D–F).

**Fig. 5.**
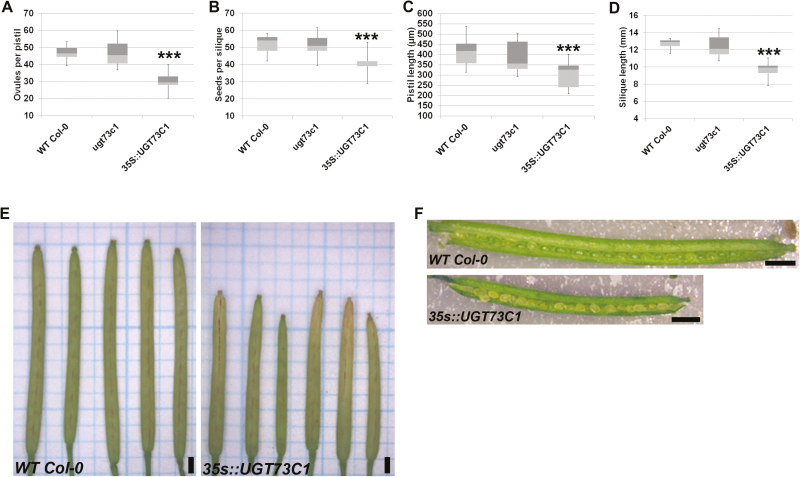
Overexpression of *UGT73C1* affects pistil and fruit elongation but not fertility. (A) Ovule number, (B) seed number, (C) pistil length, and (D) silique length in wild-type (WT) Col-0, *ugt73c1*, and *35S::UGT73C1*. The box-plots show the first (light grey) and third (dark grey) quartiles separated by the median. Error bars show the maximum and minimum values; *n*=20 pistils/siliques. Significant differences compared with WT were determined using Student’s *t*-test (***P<0.001). (E) Stereomicroscope images of wild-type and *35S::UGT73C1* mature siliques. (F) Ovules in wild-type and *35S::UGT73C1* siliques. Scale bars are 1 mm.

In parallel, the same phenotypic analysis was performed on the *UGT73C1* mutant and overexpression lines. Plants of *ugt73c1-1* did not differ from the wild-type (Fig. 5A–D). This might have been due to redundancy of the *UGT73C* family. Overexpression of *UGT73C1* caused a significant reduction in the number of ovules and seeds (Fig. 5A, B) and this variation was linked only to reductions in the length of pistils and siliques (Fig. 5C, D) since in *35S::UGT73C1* fertility was not compromised and seed-set was complete (Fig. 5E, F). In contrast, in *35S::UGT85A3* the reduction in silique length was linked both to a reduction in pistil length and to a high number of unfertilized ovules. These data suggested that UGT73C1 might be involved in processes related to pistil and fruit elongation rather than in the formation of ovules.

## Discussion

In this study we quantified CK and performed a transcriptomic analysis to investigate genes downstream of CUCs related to CK metabolic pathways. Analysis of the levels of different CK forms revealed that in *cuc2 pSTK::RNAi-CUC1* inflorescences the levels of active forms (*c*Z and iP) were reduced compared to the wild-type, while those of inactive *O*-glucosylated and *N*-glucosylated forms increased. In parallel, by comparing transcription profiles of wild-type and *cuc2 pSTK::RNAi-CUC1* pistils by RNA-seq, we obtained a list of genes that were deregulated due to the absence of CUC1 and/or CUC2,that might therefore be direct or indirect targets of CUC1/2. We selected genes that had a link to CK homeostasis and tested their possible involvement in ovule number determination. The candidates considered were *UGT73C1* and *UGT85A3*, which code for enzymes that inactivate CKs by *O*-glucosylation (Hou *et al.*, 2004) and which were up-regulated in *cuc2 pSTK::RNAi-CUC1*. Absence of *UGT85A3* positively influenced ovule number, as revealed by phenotypic analysis of two *ugt85a3* mutant lines. Indeed, *ugt85a3* plants produced more seeds without compromising their viability. This effect could be explained if *ugt85a3* mutant plants have a slight increase in active CK bases and thus have higher activity of the meristematic placenta tissue. Indeed, CKs are well known to act by maintaining cell proliferation in meristematic tissues, in particular through action on D-type cyclins (Riou-Khamlichi *et al.*, 1999; Dewitte *et al.*, 2007). It can be assumed that, in the placenta, CUCs repress the expression of CK-specific *UGT*s in order to increase the level of active CKs and decrease the glyco-conjugated inactive forms. When overexpressed, *UGT85A3* causes severe growth and fertility problems, as reported for mutants strongly impaired in CK response, such as the *cre ahk2 ahk3* CK receptor triple-mutant (Higuchi *et al.*, 2004; Nishimura *et al.*, 2004).

The second candidate, UGT73C1, seemed to play a prevalent role in pistil and fruit elongation rather than in ovule formation. Indeed, high levels of *UGT73C1* resulted in shorter pistils bearing fewer ovules, but did not compromise their fertility. It should also be noted that the two enzymes, UGT73C1 and UGT85A3, exercise their *O*-glucosylation function on different types of zeatin, which might affect different developmental processes. *t*Z and iP are the most abundant CK forms. *t*Z-types and iP-types accumulate in xylem and phloem, respectively, and generally exhibit higher activities than *c*Z (Sakakibara, 2006). According to our results, *t*Z and iP were also the most abundant active CKs in pre-fecundation inflorescences. CK *O*-glucosides have been shown to be reversibly deglycosylated by the action of β-glucosidase and therefore are thought to serve as inactive storage forms of CK (Šmehilová *et al.*, 2016). Interestingly, in the list of down-regulated genes (Supplementary Table S3) we found *BETA GLUCOSIDASE 41* (*BGLU41*, fold-change –3.992), which codes for an enzyme that may be involved in the de-glucosylation of CK *O*-glucosides. In accordance with the hypothesis proposed above, it would be interesting to investigate whether CUCs may favor the expression of *BGLU41* to increase the ‘reactivation’ of CKs.

In addition, our analysis revealed that *N*-glucosides were the most abundant form of cytokinin in the inflorescence and that their levels increased even more in the absence of *CUC1/2* (Fig. 2). Unlike *O*-glucosides, *N*-glucosides are usually resistant to β-glucosidases, making them the terminal products of irreversible deactivation. This suggests that CUCs regulate both reversible and irreversible CK inactivation. However, the putative CUC target(s) responsible for *N*-glucosylation were not identified in our RNA-seq data and remain to be discovered.

RNA-seq data do not allow discrimination between direct and indirect targets of transcription factors. CUCs have been described as positive regulators of transcription (Raman *et al.*, 2008; Galbiati *et al.*, 2013) and *UGT85A3* and *UGT73C1* were up-regulated in our dataset. Based on that, it is likely that *UGT73C1* and *UGT85A3* are indirect targets of CUCs. However, the role of a transcription factor as an activator or repressor is context-dependent, and therefore further experiments are necessary to rule out this possibility.

Judicious selection of CRISPR-Cas9 gRNAs could allow disruption of multiple *UGT* family members and unravel specific and redundant gene functions. Taken together, our results point to the role of both *UGT* genes as a link between CUCs and CK homeostasis, and highlight the importance of CUCs and CKs in seed yield determination. Furthermore, UGTs are good candidates for improved seed yield in agronomically important plants.

## Supplementary data

Supplementary data are available at *JXB* online.

Fig. S1. qRT-PCR validation of RNA-seq data for the two candidate CUC target genes *UGT85A3* and *UGT73C1*.

Fig. S2. Position of the T-DNA insertions in *UGT85A3* and *UGT73C1* mutants, and expression of *UGT85A3* and *UGT73C1* in mutant and overexpression lines.

Table S1. Total cytokinin metabolite levels in wild-type and *cuc2 pSTK::RNAi-CUC1* inflorescences.

Table S2. List of genes up-regulated in *cuc2 pSTK::RNAi-CUC1* versus wild-type pistils.

Table S3. List of genes down-regulated in *cuc2 pSTK::RNAi-CUC1* versus wild-type pistils.

Table S4. Functional categorization of biological process GO terms.

Table S5. List of primers.

Supplementary Figures and Tables
