# Supplementary material for: CUP-SHAPED COTYLEDON1 (CUC1) and CUC2 regulate cytokinin homeostasis to determine ovule number in Arabidopsis
Source: J Exp Bot. 2018 Jul 26;69(21):5169–76. doi: 10.1093/jxb/ery281 (PMC6184744; doi:10.1093/jxb/ery281)
Supplement: Supplementary Figures and Tables [file ery281_suppl_supplementary_figures-s1-s2_tables-s1-s5.pdf]

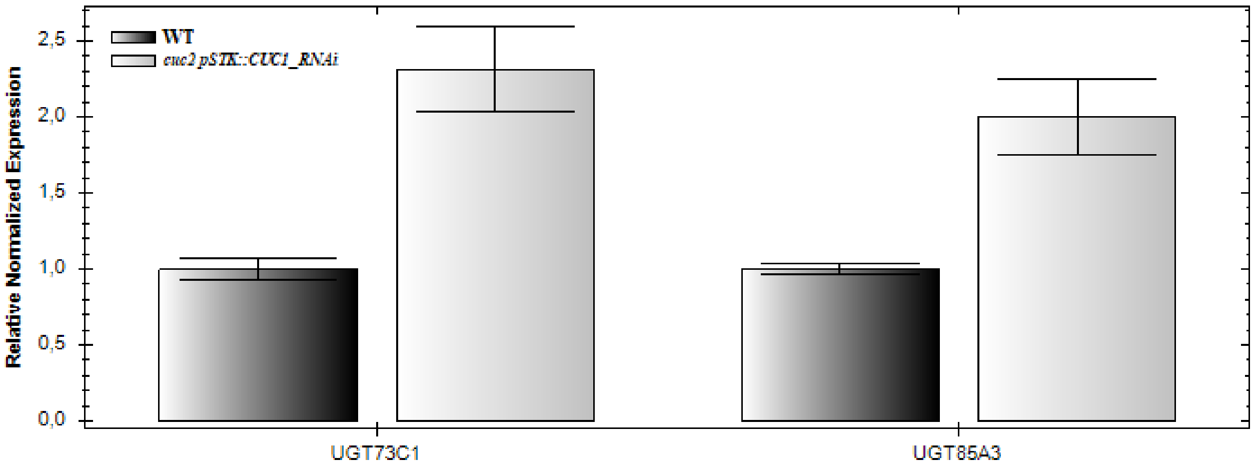

Figure S1. qRT-PCR validation of RNA-seq data for two candidate *CUC* target genes. Comparison of expression of *UGT85A3* and *UGT73C1* between wild-type and *cuc2 pSTK::CUC1_RNAi (cuc2CUC1i)* mutant pistils. Gene expression was normalized against *actin8* and *ubiquitin10*. Error bars represent the propagated error value using three technical replicates. Three biological replicates were performed with equivalent results.

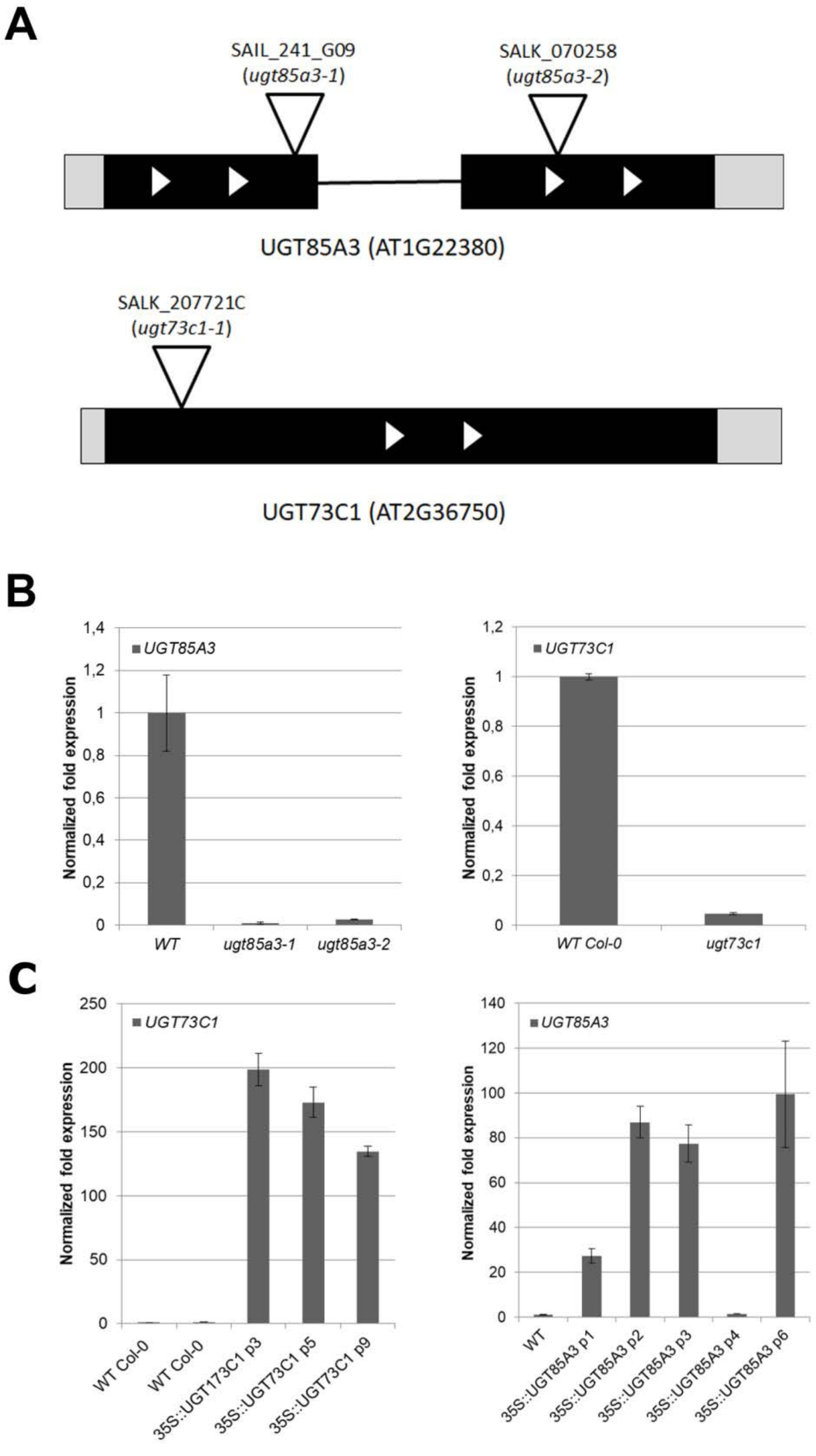

Figure S2. Position of T-DNA insertions in *UGT85A3* and *UGT73C1* mutants, and expression of *UGT85A3* and *UGT73C1* in mutant and overexpression lines. (A) Schematic representation of T-DNA insertion in *ugt85a3-1*, *ugt85a3-2* and *ugt73c1* mutants. In *ugt85a3-2* T-DNA insertion caused a rearrangement of the gene and of the T-DNA itself. (B) Real-time PCR to verify transcript accumulation in *ugt85a3-1*, *ugt85a3-2* and *ugt73c1* mutant lines. (C) Real-time PCR confirmed overexpression of *UGT73C1* in three *35S::UGT73C1* independent lines and of *UGT85A3* in four *35S::UGT85A3* independent lines. Gene expression was normalized against *actin8* and *ubiquitin10*. Error bars represent the propagated error value using three technical replicates. Three biological replicates were performed with equivalent results.

Supplemental Table S1. Total cytokinin metabolites levels (in pmol/g) in wild-type and *cuc2 pSTK::CUC1_RNAi* inflorescences.

Total cytokinin levels in pmol/g (Mean ± SD)

*Asterisks indicate statistically significant difference in mutant line (cuc2CUC1i) versus the wild type (WT) during treatment an ANOVA analysis (t-test; *, **, and *** correspond to P-values of 0.05 > p > 0.01, 0.01 > p >0.001, and p < 0.001, respectively).*

Samples	*Total CKs*	*Total CK Bases*	*Total CK Ribosides*	*otal CK Nucleotide*	*otal CK O-glucoside*	*Total CK N-glucosides*
WT	168,9 ± 23,0	▲ 7,89 ± 1,23	29,27 ± 3,06	67,32 ± 14,86	10,08 ± 1,12	▼ 54,34 ± 5,26
cuc2CUC1i	203,1 ± 8,2	▼ **5,02 ± 0,98** *	32,65 ± 4,45	82,06 ± 3,26	11,59 ± 0,84	▲ **71,78 ± 7,65** *

Cytokinin metabolites levels in pmol/g (Mean ± SD)

*Asterisks indicate statistically significant difference in mutant line (cuc2CUC1i) versus the wild type (WT) during treatment an ANOVA analysis (t-test; *, **, and *** correspond to P-values of 0.05 > p > 0.01, 0.01 > p >0.001, and p < 0.001, respectively).*

Samples	*Total tZ-types*	*tZ*	*tZR*	*tZRMP*	*tZOG*	*tZROG*	*tZ7G*	*tZ9G*
WT	66,61 ± 9,82	4,21 ± 0,46	9,67 ± 2,08	16,49 ± 5,53	1,03 ± 0,08	▼ 0,73 ± 0,16	▼ 23,13 ± 2,34	▼ 11,36 ± 1,28
cuc2CUC1i	**85,98 ± 4,51** *	3,70 ± 1,07	10,97 ± 2,52	20,60 ± 3,22	1,15 ± 0,08	▲ **0,99 ± 0,10** *	▲ **32,75 ± 4,18** *	▲ **15,81 ± 0,99** **

Samples	*Total cZ-types*	*cZ*	*cZR*	*cZRMP*	*cZOG*	*cZROG*	*cZ9G*
WT	39,08 ± 8,54	▲ 0,53 ± 0,13	4,13 ± 0,17	27,48 ± 7,82	4,53 ± 0,66	▼ 2,34 ± 0,09	0,08 ± 0,01
cuc2CUC1i	50,95 ± 7,40	▼ **0,29 ± 0,07** *	4,67 ± 1,17	38,62 ± 6,54	4,64 ± 0,28	▲ **2,64 ± 0,19** *	0,09 ± 0,02

Samples	*Total DHZ-types*	*DHZ*	*DHZR*	*DHZRMP*	*DHZOG*	*DHZROG*	*DHZ7G*	*DHZ9G*
WT	15,21 ± 3,56	0,17 ± 0,04	1,95 ± 0,43	1,20 ± 0,41	0,51 ± 0,09	▼ 0,95 ± 0,31	9,48 ± 2,23	0,95 ± 0,29
cuc2CUC1i	19,94 ± 1,61	*< LOD*	2,44 ± 0,66	1,56 ± 0,40	0,70 ± 0,21	▲ **1,45 ± 0,14** *	12,48 ± 2,18	1,31 ± 0,15

Samples	*Total iP-types*	*iP*	*iPR*	*iPRMP*	*iP7G*	*iP9G*
WT	47,99 ± 3,30	▲ 2,98 ± 0,90	13,52 ± 0,56	22,15 ± 2,71	9,08 ± 1,31	0,26 ± 0,05
cuc2CUC1i	46,22 ± 7,46	▼ **1,03 ± 0,33** *	14,57 ± 4,58	21,29 ± 2,59	9,10 ± 1,07	0,24 ± 0,02

Supplemental Table 2. Up-regulated genes in *cuc2 pSTK::RNAi-CUC1* pistils.

Locus	Gene name/ Splicing form	Fold change	PValue	Gene Description
AT5G44785	OSB3.2	313.683	0.001	organellar single-stranded DNA binding protein 3;
AT1G53910	RAP2.12.1	226.247	0	Encodes a member of the ERF subfamily B-2 of ERF/AP2 transcription factor family.
AT1G25350	OVA9.2	216.409	0.005	glutamine-tRNA ligase, putative / glutaminyl-tRNA synthetase, putative / GlnRS;
AT1G31930	XLG3.3	91.869	0.008	Encodes XLG3 that shows significant similarity to the G protein alpha subunit in its C terminal region
AT2G31865	PARG2.2	79.502	0.002	poly(ADP-ribose) glycohydrolase 2;
AT3G04770	RPSAb.2	78.944	0	40s ribosomal protein SA B;
AT2G38020	VCL1.2	53.724	0.048	necessary for proper vacuole formation and morphogenesis in Arabidopsis
AT3G61415	SK21.2	44.042	0.008	SKP1-like 21;
AT1G02880	TPK1.1	40.805	0	Encodes a thiamine pyrophosphokinase capable of producing thiamine pyrophosphate from free thiamine.
AT5G53050	AT5G53050.3	40.151	0.002	alpha/beta-Hydrolases superfamily protein;
AT5G20390	AT5G20390.1	37.047	0.049	Glycosyl hydrolase superfamily protein;
AT5G53050	AT5G53050.2	32.27	0	alpha/beta-Hydrolases superfamily protein;
AT4G20380	AT4G20380.6	24.242	0.021	LSD1 monitors a superoxide-dependent signal and negatively regulates a plant cell death pathway.
AT1G07890	APX1.2	22.962	0.01	Encodes a cytosolic ascorbate peroxidase APX1.
AT3G16230	AT3G16230.1	20.895	0.003	Putative eukaryotic LigT;
AT3G21570	AT3G21570.1	20.672	0.002	proline-rich nuclear receptor coactivator;
AT5G08080	SYP132.3	20.265	0	member of SYP13 Gene Family
AT4G37830	AT4G37830.2	18.723	0.002	cytochrome c oxidase-like protein;
AT1G18390	AT1G18390.2	16.877	0.01	Serine/Threonine kinase family catalytic domain protein;
AT4G27790	AT4G27790.1	16.408	0.024	Calcium-binding EF hand family protein;
AT1G29450	AT1G29450.1	15.226	0.002	SAUR-like auxin-responsive protein family;
AT1G71860	PTP1.2	15.144	0	Encodes a protein with tyrosine phosphatase activity
AT1G28100	AT1G28100.2	15.07	0.002	hypothetical protein;
AT4G16360	AT4G16360.1	14.186	0	5'-AMP-activated protein kinase beta-2 subunit protein
AT1G54360	TAF6B.5	13.621	0.005	Encodes one of two Arabidopsis proteins with significant similarity to the histone fold TBP-associated factor TAF6.
AT5G59560	SRR1.2	12.957	0.048	Normal function of the protein is required for normal oscillator function during circadian rhythm.
AT2G43490	AT2G43490.1	12.856	0.027	Ypt/Rab-GAP domain of gyp1p superfamily protein;

AT2G34310	AT2G34310.3	12.708	0.008	hypothetical protein;
AT5G03435	AT5G03435.1	12.635	0.004	Ca2+dependent plant phosphoribosyltransferase family protein;
AT1G26290	AT1G26290.1	12.521	0.005	hypothetical protein;
AT5G56150	UBC30.2	11.616	0.005	ubiquitin-conjugating enzyme 30;
AT5G23870	AT5G23870.1	11.457	0.008	Encodes a pectin acetylesterase that removes cell wall acetate associated with pectin formation in Arabidopsis leaves.
AT5G08079	SYP132.1	11.381	0	member of SYP13 gene Family
AT5G17910	AT5G17910.2	11.204	0.018	cardiomyopathy-associated protein;
AT2G48100	AT2G48100.3	11.162	0.015	Exonuclease family protein;
AT5G18240	MYR1.1	11.011	0.028	Encodes MYR1 (MYR1).
AT5G01225	AT5G01225.1	10.81	0.02	josephin-like protein;
AT5G20400	AT5G20400.1	10.415	0.02	Encodes a protein whose sequence is similar to flavanone 3 hydroxylase from Malus.
AT1G75250	RL6.1	10.343	0.005	RAD-like 6
AT2G45150	CDS4.1	10.25	0.015	cytidinediphosphate diacylglycerol synthase 4;
AT2G45403	AT2G45403.1	9.801	0.016	hypothetical protein;
AT3G49680	BCAT3.2	9.685	0.019	Encodes a chloroplast branched-chain amino acid aminotransferase.
AT5G62640	AT5G62640.1	9.611	0	Nuclear targeted protein involved in flowering time regulation that affects flowering time independent of FLC
AT5G66390	AT5G66390.1	9.465	0.01	Encodes a peroxidase that is involved in lignin biosynthesis.
AT2G45800	AT2G45800.2	9.421	0.014	Encodes a member of the Arabidopsis LIM proteins: a family of actin bundlers with distinct expression patterns.
AT3G15604	AT3G15604.1	9.338	0.037	hypothetical protein;
AT3G06260	GATL4.1	9.115	0.003	Encodes a protein with putative galacturonosyltransferase activity.
AT3G09410	AT3G09410.2	9	0.037	Pectinacetylesterase family protein;
AT5G46880	HB-7	8.868	0.022	homeobox-7;
AT5G65080	MAF5.1	8.787	0.048	Is upregulated during vernalization and regulates flowering time. Encodes MADS-domain protein.
AT1G14990	AT1G14990.1	8.666	0.035	transmembrane protein;
AT3G28270	AT3G28270.2	8.641	0.01	Peripheral membrane protein associated with endomembranes and plasmamembrane.
AT5G14930	SAG101.3	8.405	0.027	encodes an acyl hydrolase involved in senescence .
AT5G14250	COP13.2	8.274	0.019	Encodes subunit 3 of the COP9 signalosome.
AT3G09710	IQD1.1	8.032	0.048	Ca(2+)-dependent calmodulin-binding protein.
AT2G34186	AT2G34186.1	7.847	0.019	hypothetical protein;
AT2G14120	DRP3B.3	7.806	0.02	Encodes a dynamin related protein. DRPs are self-assembling GTPasse involved in fission and fusion of membranes.
AT5G66400	RAB18	7.803	0.011	Belongs to the dehydrin protein family.

AT1G78915	AT1G78915.3	7.802	0.05	Tetratricopeptide repeat (TPR)-like superfamily protein.
AT4G21540	SPHK1.3	7.75	0.023	Encodes a sphingosine kinase. Involved in guard cell ABA signalling and seed germination.
AT5G61660	AT5G61660.1	7.731	0.007	glycine-rich protein;
AT2G20530	PHB6.2	7.592	0.041	prohibitin 6;
AT2G26430	RCY1.3	7.577	0.015	Encodes an ania-6a type arginine-rich cyclin which confers tolerance to LiCl and NaCl when expressed in yeast.
AT1G53830	PME2.1	7.267	0.018	encodes a pectin methylesterase
AT1G14240	AT1G14240.4	7.241	0.021	GDA1/CD39 nucleoside phosphatase family protein;
AT4G31990	ASP5.3	7.15	0.022	Encodes a plastid-localized aspartate aminotransferase.
AT4G19690	IRT1.1	7.067	0.017	The gene encodes Fe2+ transporter protein. It is a member of the Zrt/Irt-like protein (ZIP) family of transporters.
AT2G45740	PEX11D.3	6.814	0.044	member of the peroxin11 (PEX11) gene family, integral to peroxisome membrane, controls peroxisome proliferation.
AT1G23970	AT1G23970.1	6.783	0.04	hypothetical protein (DUF626);
AT1G06810	AT1G06810.1	6.76	0.043	endonuclease/glycosyl hydrolase;
AT2G28610	PRS.1	6.709	0.023	Encodes a homeodomain containing protein that regulates lateral axis-dependent development of Arabidopsis flowers.
AT1G06970	CHX14.1	6.693	0.013	member of Putative Na+/H+ antiporter family
AT2G14680	MEE13.1	6.518	0.034	myosin heavy chain-like protein;
AT4G31990	ASP5.1	6.47	0.028	Encodes a plastid-localized aspartate aminotransferase.
AT3G56580	AT3G56580.2	6.417	0.027	Encodes a functional E3 ubiquitin ligase.
AT1G65960	GAD2.2	6.321	0.008	glutamate decarboxylase (GAD2).
AT4G23960	AT4G23960.1	6.258	0.023	F-box family protein;
AT3G04030	AT3G04030.1	6.123	0.002	Homeodomain-like superfamily protein;
AT4G31180	AT4G31180.2	6.066	0.029	The IBI1 gene encodes an aspartyl tRNA synthetase (AspRS).
AT1G48020	PMEI1.1	5.973	0.039	Pectin methylesterase inhibitor AtPMEI1. Inactivates AtPPME1 in vitro. Localized to pollen tube cell tip.
AT1G76955	AT1G76955.1	5.703	0.036	Expressed protein;
AT2G31500	CPK24.1	5.65	0.042	member of Calcium Dependent Protein Kinase
AT3G15490	AT3G15490.1	5.43	0.042	Regulator of Vps4 activity in the MVB pathway protein;
AT3G23860	AT3G23860.1	5.373	0.042	Encodes a GTP-binding related protein
AT1G56280	DI19.1	5.231	0.049	Encodes a gene whose transcript level in root and leaves increases to progressive drought stress.
AT2G18180	AT2G18180.1	5.228	0.016	Sec14p-like phosphatidylinositol transfer family protein;
AT3G49430	SRp34a.2	5.22	0.041	SER/ARG-rich protein 34A;
AT4G22750	AT4G22750.1	5.129	0.01	protein S-acyltransferase
AT4G12410	AT4G12410.1	5.107	0.006	SAUR-like auxin-responsive protein family;

AT2G20830	AT2G20830.1	5.012	0.032	folic acid binding / transferase;
AT5G17760	AT5G17760.2	5.005	0.036	P-loop containing nucleoside triphosphate hydrolases superfamily protein;
AT2G38880	NF-YB1.3	4.982	0.019	Encodes a transcription factor from the nuclear factor Y (NF-Y) family, AtNF-YB1.
AT4G11350	AT4G11350.1	4.875	0.015	transferring glycosyl group transferase (DUF604);
AT2G47090	AT2G47090.1	4.671	0.012	zinc ion binding/nucleic acid binding protein;
AT3G06778	AT3G06778.1	4.611	0.002	Chaperone DnaJ-domain superfamily protein;
AT5G13750	ZIFL1.3	4.534	0.01	zinc induced facilitator-like 1;
AT2G01660	PDLP6.1	4.466	0.048	Encodes a plasmodesmal protein
AT5G42250	AT5G42250.1	4.399	0.016	Zinc-binding alcohol dehydrogenase family protein
AT5G07770	AT5G07770.2	4.322	0.04	Actin-binding FH2 protein;
AT5G26030	FC1.3	4.28	0.035	Encodes ferrochelatase I located in plastids.
AT2G17220	AT2G17220.1	4.276	0.002	Encodes a putative serine/threonine-specific protein kinase kin3. Protein is N-myristoylated.
AT1G44542	AT1G44542.1	4.167	0.014	Cyclase family protein;
AT3G48860	AT3G48860.2	4.14	0.018	coiled-coil protein;
AT4G22140	EBS.2	4.14	0.017	PHD finger family protein / bromo-adjacent homology (BAH) domain-containing protein;
AT4G33430	BAK1.1	4.108	0.023	Leu-rich receptor Serine/threonine protein kinase. Component of BR signaling.
AT3G49680	BCAT-1.2	4.066	0.031	Encodes a chloroplast branched-chain amino acid aminotransferase.
AT1G14100	FUT8.1	4.026	0.042	member of Glycosyltransferase Family- 37. FUT8 was previously associated to AT1G14110
AT1G75060	AT1G75060.2	4.019	0.028	histone deacetylase complex subunit;
AT2G07719	AT2G07719.1	3.9	0.024	Putative membrane lipoprotein;
AT1G01980	ATSEC1A.1	3.883	0.024	member of Reticuline oxidase-like family
AT2G38152	AT2G38152.1	3.877	0	alpha 1,4-glycosyltransferase family protein;
AT1G67490	GCS1.1	3.814	0.038	Encodes an alpha-glucosidase I enzyme that catalyzes the first step in N-linked glycan processing.
AT3G01080	WRKY58.1	3.749	0.031	member of WRKY Transcription Factor; Group I
AT1G02840	SR1	3.747	0.008	SR34/SR1 is a plant homologue of the human general/alternative splicing factor SF2/ASF
AT5G58310	MES18.1	3.733	0.042	Encodes a protein shown to have methyl IAA esterase activity in vitro.
AT5G43310	AT5G43310.4	3.72	0	COP1-interacting protein-like protein;
AT5G09690	MGT7.3	3.714	0.014	Transmembrane magnesium transporter.
AT3G13140	AT3G13140.1	3.645	0.039	hydroxyproline-rich glycoprotein family protein;
AT5G36550	AT5G36550.1	3.623	0.05	Encodes a ECA1 gametogenesis related family protein
AT1G58280	AT1G58280.1	3.615	0.044	Phosphoglycerate mutase family protein;

AT3G13784	CWINV5.1	3.567	0.018	cell wall invertase 5;
AT3G13660	AT3G13660.1	3.423	0.044	Disease resistance-responsive (dirigent-like protein) family protein;
AT5G63450	CYP94B1.1	3.421	0.036	member of CYP94B
AT2G38170	CAX1.2	3.408	0.007	Encodes a high affinity vacuolar calcium antiporter.
AT1G20190	EXPA11.1	3.405	0.002	member of Alpha-Expansin Gene Family.
AT3G01085	AT3G01085.1	3.363	0.046	Protein kinase superfamily protein;
AT1G22380	UGT85A3.1	3.35	0.004	Encodes a putative UDP-glucosyl transferase.
AT4G35100	PIP3.1	3.345	0.016	a member of the plasma membrane intrinsic protein PIP. functions as aquaporin. Salt-stress-inducible MIP
AT5G28237	AT5G28237.1	3.343	0.032	Pyridoxal-5-phosphate-dependent enzyme family protein;
AT1G30135	JAZ8.1	3.325	0.029	jasmonate-zim-domain protein 8;
AT5G67340	AT5G67340.1	3.317	0.004	ARM repeat superfamily protein;
AT2G04066	AT2G04066.1	3.316	0.017	MATE efflux family protein;
AT5G43200	AT5G43200.1	3.182	0.049	Zinc finger, C3HC4 type (RING finger) family protein;
AT2G25185	AT2G25185.1	3.168	0.048	Encodes a defensin-like (DEFL) family protein.
AT1G80320	AT1G80320.1	3.129	0.032	2-oxoglutarate (2OG) and Fe(II)-dependent oxygenase superfamily protein;
AT5G02470	DPA.1	3.091	0.034	core cell cycle genes
AT3G10815	AT3G10815.1	3.058	0.028	RING/U-box superfamily protein;
AT4G38910	BPC5.2	3.004	0.002	Encodes a basic pentacysteine protein.
AT3G47350	HSD2.2	2.988	0.009	Encodes a putative hydroxysteroid dehydrogenase (HSD).
AT4G39370	UBP27.1	2.984	0.011	Encodes a ubiquitin-specific protease.
AT5G55040	AT5G55040.1	2.931	0.05	DNA-binding bromodomain-containing protein;
AT3G20210	DELTA-VPE.1	2.91	0.024	Encodes a vacuolar processing enzyme with caspase-1-like activity
AT2G36750	UGT73C1.1	2.904	0.062	UDP-glucosyl transferase 73C1;
AT5G59330	AT5G59330.1	2.893	0.036	Bifunctional inhibitor/lipid-transfer protein/seed storage 2S albumin superfamily protein;
AT5G65640	bHLH093.2	2.888	0.032	bHLH093/NFL encodes a bHLH transcription factor involved in GA mediated control of flowering time.
AT2G43920	HOL2.1	2.853	0.037	S-adenosyl-L-methionine-dependent methyltransferases superfamily protein;
AT5G02170	AT5G02170.2	2.838	0.045	Transmembrane amino acid transporter family protein;
AT3G03670	AT3G03670.1	2.828	0.032	Peroxidase superfamily protein;
AT4G21620	AT4G21620.2	2.818	0.007	glycine-rich protein;
AT4G23690	AT4G23690.1	2.794	0.008	Encodes a homodimeric all-beta dirigent protein in the superfamily of calycins.

AT1G34760	GRF11.2	2.784	0.012	Encodes a 14-3-3 protein. Binds H+-ATPase in response to blue light.
AT4G25430	AT4G25430.1	2.723	0.006	hypothetical protein;
AT5G09410	EICBP.B.3	2.723	0.034	calmodulin-binding protein
AT1G70480	AT1G70480.2	2.7	0.026	OBP32pep, putative (DUF220);
AT1G30280	AT1G30280.1	2.67	0.045	Chaperone DnaJ-domain superfamily protein;
AT1G70950	AT1G70950.1	2.648	0.009	TPX2 (targeting protein for Xklp2) protein family;
AT3G27809	AT3G27809.1	2.614	0.028	hypothetical protein;
AT1G64405	AT1G64405.1	2.61	0.014	hypothetical protein;
AT5G48950	AT5G48950.1	2.553	0.037	Encodes one of the two functional DHNA-CoA (1,4-dihydroxy-2-naphthoyl-CoA) thioesterases found in Arabidopsis.
AT2G43920	HOL2.2	2.532	0.036	S-adenosyl-L-methionine-dependent methyltransferases superfamily protein;
AT4G38510	AT4G38510.1	2.531	0.041	One of three genes encoding the vacuolar ATP synthase subunit B1.
AT3G53460	CP29.3	2.511	0.022	Encodes a nuclear gene with a consensus RNA-binding domain that is localized to the chloroplast.
AT5G38900	AT5G38900.1	2.505	0.005	Thioredoxin superfamily protein;
AT5G20870	AT5G20870.1	2.504	0.021	O-Glycosyl hydrolases family 17 protein;
AT1G02470	AT1G02470.1	2.492	0.012	Polyketide cyclase/dehydrase and lipid transport superfamily protein;
AT3G14380	AT3G14380.1	2.487	0.043	Uncharacterized protein family (UPF0497);
AT5G25110	CIPK25.1	2.452	0.022	member of AtCIPKs
AT5G20470	AT5G20470.1	2.413	0.012	Encodes a headless derivative of myosin XI-K
AT3G06020	AT3G06020.1	2.41	0.024	A member of the FAF family proteins encoded by the FANTASTIC FOUR (FAF) genes
AT4G11290	AT4G11290.1	2.383	0.017	Peroxidase superfamily protein;
AT5G50950	FUM2.1	2.382	0.049	Encodes a fumarase enzyme present in the cytosol.
AT3G50120	AT3G50120.1	2.348	0.013	transmembrane protein, putative (DUF247);
AT1G66240	ATX1	2.34	0.044	homolog of anti-oxidant 1;
AT1G65550	AT1G65550.1	2.335	0.038	Xanthine/uracil permease family protein;
AT1G29230	CIPK18.1	2.294	0.013	Encodes a member of the SNF1-related kinase (SnRK) gene family (SnRK3.20)
AT1G05450	AT1G05450.1	2.274	0.027	Encodes a Protease inhibitor/seed storage/LTP family protein
AT4G22540	ORP2A.4	2.268	0.011	OSBP(oxysterol binding protein)-related protein 2A;
AT1G20310	AT1G20310.1	2.267	0.009	syringolide-induced protein;
AT5G58580	ATL63.1	2.264	0.012	Encodes a functional E3 ligase that is involved in membrane trafficking and regulation of salt stress responses.
AT4G13266	AT4G13266.1	2.259	0.023	ankyrin repeat protein;
AT5G44480	DUR.1	2.247	0.024	UDP Glucose Epimerase. Mutant has Altered lateral root;

AT3G27810	MYB21.1	2.238	0.04	Encodes a member of the R2R3-MYB transcription factor gene family.
AT2G39890	PROT1.2	2.234	0.004	Encodes a proline transporter with affinity for gly betaine, proline and GABA. Protein is expressed in the phloem.
AT1G53470	MSL4.1	2.23	0.05	mechanosensitive channel of small conductance-like 4;
AT1G65450	AT1G65450.2	2.23	0.038	Contains dual transcription units and alternative splicing that could rescue the sterility defect of glc mutants.
AT1G23140	AT1G23140.1	2.227	0.024	Expression is upregulated in the shoot of cax1/cax3 mutant.
AT5G42770	AT5G42770.2	2.22	0.05	Maf-like protein;
AT2G24450	FLA3.1	2.216	0.03	FASCICLIN-like arabinogalactan protein 3 precursor;
AT5G43230	AT5G43230.1	2.188	0.034	EEIG1/EHBP1 protein amino-terminal domain protein;
AT5G11400	AT5G11400.2	2.167	0.022	Protein kinase superfamily protein;
AT5G53420	AT5G53420.2	2.149	0.027	CCT motif family protein;
AT5G38320	AT5G38320.2	2.096	0.008	hypothetical protein;
AT2G34600	JAZ7.1	2.069	0.002	jasmonate-zim-domain protein 7;
AT2G44260	AT2G44260.1	2.061	0.045	DUF946 family protein (DUF946);
AT5G23820	AT5G23820.1	2.023	0.032	ML3 can be modified by NEDD8 and ubiquitin.
AT5G09970	CYP78A7.1	2.022	0.027	member of CYP78A
AT2G33850	AT2G33850.1	2.021	0.036	E6-like protein;
AT3G55646	AT3G55646.1	2	0.002	TPRXL;
AT1G54575	AT1G54575.1	1.986	0.021	hypothetical protein;
AT1G06620	AT1G06620.1	1.974	0.012	encodes a protein whose sequence is similar to a 2-oxoglutarate-dependent dioxygenase.
AT3G48510	AT3G48510.1	1.909	0.018	AtIII18x5-like protein;
AT1G32540	LOL1.1	1.876	0.017	Encodes a protein with 3 plant-specific zinc finger domains that acts as a positive regulator of cell death.
AT3G12000	AT3G12000.1	1.862	0.023	S-locus related protein SLR1, putative (S1);
AT4G34750	AT4G34750.1	1.857	0.043	SAUR-like auxin-responsive protein family;
AT4G25000	AMY1.1	1.845	0.043	Predicted to be secreted protein based on signalP prediction. Involved in starch mobilization.
AT5G59810	SBT5.4.1	1.845	0.026	Subtilase family protein;
AT3G26115	AT3G26115.2	1.842	0.046	Pyridoxal-5-phosphate-dependent enzyme family protein;
AT2G22090	UBA1A.2	1.829	0.017	encodes a nuclear protein that binds to RNA with a specificity for oligouridylates in vitro
AT1G77700	AT1G77700.1	1.8	0.028	Pathogenesis-related thaumatin superfamily protein;
AT2G28650	EXO70H8.1	1.796	0.003	A member of EXO70 gene family, putative exocyst subunits, conserved in land plants.
AT3G15605	AT3G15605.2	1.764	0.029	nucleic acid binding protein;
AT5G11200	AT5G11200.3	1.764	0.033	ENcodes an ATP-dependent RNA helicase that localizes predominantly to euchromatic regions.

AT3G01860	AT3G01860.1	1.752	0.041	hypothetical protein;
AT2G20700	LLG2.1	1.746	0.025	LORELEI-LIKE-GPI ANCHORED PROTEIN 2;
AT2G19893	AT2G19893.1	1.744	0.017	Encodes a defensin-like (DEFL) family protein.
AT4G31720	TAFII15.1	1.743	0.03	Arabidopsis thaliana putative TBP-associated 15 kDa subunit protein (TAFII15)
AT1G59850	AT1G59850.1	1.735	0.041	ARM repeat superfamily protein;
AT4G30110	HMA2.1	1.724	0.02	encodes a protein similar to Zn-ATPase, a P1B-type ATPases transport zinc
AT1G80120	AT1G80120.1	1.718	0.03	LURP-one-like protein (DUF567);
AT1G28200	FIP1.1	1.71	0.007	VirF-interacting protein FIP1
AT3G28917	MIF2.1	1.71	0.008	mini zinc finger 2;
AT5G43500	ARP9.1	1.702	0.029	encodes a protein whose sequence is similar to actin-related proteins (ARPs) in other organisms.
AT1G06520	GPAT1.1	1.674	0.024	sn-glycerol-3-phosphate 2-O-acyltransferase. Expressed in flower buds and siliques.
AT1G23730	BCA3.1	1.666	0.003	beta carbonic anhydrase 3;
AT1G20070	AT1G20070.1	1.652	0.032	hypothetical protein;
AT2G31450	ATNTH1.2	1.651	0.014	DNA glycosylase superfamily protein;
AT3G49055	AT3G49055.1	1.641	0.003	ATP-binding protein;
AT3G47560	AT3G47560.1	1.636	0.014	alpha/beta-Hydrolases superfamily protein;
AT1G35830	AT1G35830.1	1.635	0.022	VQ motif-containing protein;
AT1G77880	AT1G77880.2	1.632	0.047	Galactose oxidase/kelch repeat superfamily protein;
AT1G62700	ANAC026.1	1.624	0.009	Encodes a NAC-domain transcription factor. Expressed in the vascular tissue.
AT4G25050	ACP4.1	1.624	0.048	encodes an acyl carrier protein predominantly expressed in leaves. Gene expression is upregulated by light.
AT3G07525	ATG10.1	1.619	0.038	Encodes an E1 ligase involved in autophagic vesicle formation.
AT1G02520	PGP11.1	1.613	0.039	Encodes an ATP-binding cassette (ABC) transporter. Expressed in the vascular tissue of primary stem.
AT1G47270	TLP6.1	1.601	0.016	Member of TLP family
AT1G31830	AT1G31830.2	1.596	0.028	Encodes POLYAMINE UPTAKE TRANSPORTER 2, an amino acid permease family protein.
AT1G48760	delta-ADR.1	1.595	0.006	Encodes the putative delta subunit of the AP(adaptor protein)-3 complex and plays a role in vacuolar function.
AT4G27440	PORB.1	1.585	0.032	light-dependent NADPH:protochlorophyllide oxidoreductase B
AT5G02420	AT5G02420.1	1.585	0.007	cyclin-dependent kinase inhibitor SMR3-like protein;
AT3G26120	TEL1.1	1.574	0.008	Similar to terminal ear1 in Zea mays. A member of mei2-like gene family.
AT1G04040	AT1G04040.1	1.555	0.018	HAD superfamily, subfamily IIIB acid phosphatase;
AT3G07760	AT3G07760.2	1.555	0.041	Sterile alpha motif (SAM) domain-containing protein;
AT1G12880	NUDT12.1	1.544	0.034	nudix hydrolase homolog 12;

AT5G50520	AT5G50520.1	1.53	0.043	Major facilitator superfamily protein;
AT5G10800	AT5G10800.1	1.529	0.001	RNA recognition motif (RRM)-containing protein;
AT4G17905	ATL4H.1	1.517	0.009	Putative RING-H2 finger protein ATL4H.
AT5G65530	AT5G65530.1	1.516	0.011	Encodes a protein kinase involved in mediating resistance to fungi and also trichome branch number.
AT1G31580	ECS1.1	1.515	0.047	Encodes cell wall protein.
AT1G43170	RP1	1.514	0.015	Encodes a cytoplasmic ribosomal protein.
AT4G02590	UNE12.3	1.512	0.028	basic helix-loop-helix (bHLH) DNA-binding superfamily protein;
AT5G14550	AT5G14550.1	1.501	0.025	Core-2/I-branching beta-1,6-N-acetylglucosaminyltransferase family protein;

Supplemental Table 3. Down-regulated genes in *cuc2 pSTK::RNAi-CUC1* pistils.

Locus	Gene name/ Splicing form	Fold change	PValue	Gene Description
AT4G33200	XI-I.1	-680.472	0.009	member of Myosin-like proteins
AT4G38230	CPK26.1	-401.463	0.012	member of Calcium Dependent Protein Kinase
AT5G42540	XRN2.1	-238.294	0.023	In Arabidopsis XRN2 acts as a suppressor of posttranscriptional gene silencing.
AT5G43310	AT5G43310.1	-65.535	0.003	COP1-interacting protein-like protein
AT5G43310	AT5G43310.3	-51.981	0.002	COP1-interacting protein-like protein
AT1G31290	AGO3.1	-50.583	0.028	ARGONAUTE 3
AT5G43310	AT5G43310.2	-41.723	0.001	COP1-interacting protein-like protein
AT4G00165	AT4G00165.2	-37.222	0.039	Bifunctional inhibitor/lipid-transfer protein/seed storage 2S albumin superfamily protein
AT3G25585	AAPT2.1	-18.209	0.001	aminoalcoholphosphotransferase (AAPT2) mRNA, complete cds
AT4G00420	AT4G00420.1	-17.913	0.005	Double-stranded RNA-binding domain (DsRBD)-containing protein
AT1G09930	OPT2.1	-17.124	0.037	oligopeptide transporter
AT4G39370	UBP27.2	-14.115	0.011	Encodes a ubiquitin-specific protease.
AT5G06250	DPA4.2	-13.617	0.003	DEVELOPMENT-RELATED PCG TARGET IN THE APEX 4 (DPA4),AP2/B3-like transcriptional factor family protein
AT1G32850	UBP11.1	-12.725	0.05	ubiquitin-specific protease 11
AT4G39370	UBP27.3	-12.152	0.016	Encodes a ubiquitin-specific protease.
AT2G22060	AT2G22060.1	-11.511	0.014	galactose oxidase/kelch repeat protein
AT1G01190	CYP78A8.1	-11.403	0.022	member of CYP78A
AT2G17220	AT2G17220.2	-11.272	0.003	Encodes a putative serine/threonine-specific protein kinase kin3. Protein is N-myristoylated.
AT4G38880	ASE3.1	-11.066	0.037	GLN PHOSPHORIBOSYL PYROPHOSPHATE AMIDOTRANSFERASE 2
AT4G23260	CRK18.2	-11.057	0.007	Encodes a cysteine-rich receptor-like protein kinase.
AT2G17690	SDC.1	-10.966	0.008	Encodes an F-box domain containing protein that is regulated by non-CG DNA methylation.
AT4G32540	YUC1.1	-9.188	0.022	Mutant has elevated levels of free IAA in dominant mutant allele; Flavin Monooxygenase-Like Enzyme; Auxin Biosynthesis
AT1G75170	AT1G75170.3	-9.064	0.001	Sec14p-like phosphatidylinositol transfer family protein
AT3G01175	AT3G01175.1	-8.996	0.039	transmembrane protein
AT3G19720	ARC5.1	-7.913	0.045	Encodes a novel chloroplast division protein.
AT3G09922	IPS1.1	-7.852	0.012	Encodes a gene product whose expression is responsive to both phosphate (Pi) and phosphite (Phi) in both roots and shoots.
AT1G45545	AT1G45545.1	-7.739	0.035	WEAK CHLOROPLAST MOVEMENT UNDER BLUE LIGHT-like protein (DUF827)
AT5G13360	AT5G13360.3	-7.61	0.046	Auxin-responsive GH3 family protein

AT3G51700	AT3G51700.1	-7.542	0.026	PIF1 helicase
AT2G31770	ARI9.1	-7.041	0.036	RING/U-box superfamily protein
AT3G09620	AT3G09620.1	-6.878	0.02	P-loop containing nucleoside triphosphate hydrolases superfamily protein
AT1G63550	AT1G63550.1	-6.819	0.036	Receptor-like protein kinase-related family protein
AT1G79050	AT1G79050.1	-6.699	0.024	recA DNA recombination family protein
AT1G63760	ARI16.1	-6.515	0.044	pseudogene of RING/U-box superfamily protein
AT5G07880	SNAP29.1	-6.437	0.034	member of mammalian SNAP25 Gene Family, a type of SNARE proteins with two chains.
AT5G04080	AT5G04080.2	-6.259	0.019	cysteine-rich TM module stress tolerance protein
AT5G07040	AT5G07040.1	-5.633	0.032	RING/U-box superfamily protein
AT5G51040	AT5G51040.3	-5.526	0.026	Encodes succinate dehydrogenase assembly factor 2 (SDHAF2), a low abundance mitochondrial protein
AT1G04945	AT1G04945.2	-5.324	0.035	HIT-type Zinc finger family protein
AT1G69320	CLE10.1	-5.119	0.021	Member of a large family of putative ligands homologous to the Clavata3 gene. Consists of a single exon.
AT1G62080	AT1G62080.1	-4.832	0.027	Encodes a member of a mucilage protein family. Predicted in silico to be glycosylated.
AT2G31940	AT2G31940.1	-4.789	0.02	oxidoreductase/transition metal ion-binding protein
AT1G55580	LAS.1	-4.723	0.038	Encodes a member of the GRAS family of putative transcriptional regulators.
AT1G79990	AT1G79990.4	-4.709	0.042	coatomer subunit beta-2
AT2G41120	AT2G41120.1	-4.5	0.023	DUF309 domain protein
AT5G53950	CUC2.1	-4.374	0.005	Transcriptional activator of the NAC gene family.
AT5G57520	ZFP2.1	-4.222	0.032	Encodes a zinc finger protein containing only a single zinc finger.
AT2G13650	GONST1.2	-4.205	0.023	Encodes a Golgi-localized GDP-mannose transporter. It can transport ADP-glucose in vitro.
AT2G46500	PI4K GAMMA 4.1	-4.165	0.013	Phosphoinositide kinase which undergo autophosphorylation and phosphorylate serine/threonine residues of protein substrates.
AT2G04039	AT2G04039.1	-4.111	0.031	NdhV is loosely associated with the NDH complex and is required for stabilizing NDH subcomplexes A and E.
AT2G40710	AT2G40710.1	-4.096	0.015	hemolysin-III related integral membrane protein
AT5G54570	BGLU41.1	-3.992	0.016	beta glucosidase 41
AT3G61340	AT3G61340.1	-3.903	0.022	F-box and associated interaction domains-containing protein
AT1G05610	APS2.1	-3.89	0.038	Encodes the small subunit of ADP-glucose pyrophosphorylase.
AT4G02690	AT4G02690.1	-3.784	0.021	Bax inhibitor-1 family protein
AT5G35620	LSP1.2	-3.773	0.018	Cap-binding protein, binds to the 5' cap structure of nuclear-encoded mRNAs. Mutant is resistant to potyvirus infection.
AT2G35940	BLH1.2	-3.73	0.047	Encodes a member of the BEL-like homeodomain protein family.
AT5G59890	ADF4.2	-3.646	0.05	Actin depolymerizing factor 4 (ADF4) mRNA, complete cds

AT2G18500	OFP7.1	-3.642	0.017	ovate family protein 7
AT5G53050	AT5G53050.1	-3.583	0.007	alpha/beta-Hydrolases superfamily protein
AT4G27250	AT4G27250.2	-3.541	0.048	NAD(P)-binding Rossmann-fold superfamily protein
AT1G60190	AT1G60190.1	-3.507	0.039	Encodes PUB19, a plant U-box armadillo repeat protein. Involved in salt inhibition of germination together with PUB18.
AT3G62000	AT3G62000.1	-3.506	0.036	S-adenosyl-L-methionine-dependent methyltransferases superfamily protein
AT3G20395	AT3G20395.1	-3.377	0.05	RING/U-box superfamily protein
AT1G64940	CYP89A6.1	-3.316	0.028	member of CYP89A
AT5G08080	SYP132.2	-3.286	0	member of SYP13 Gene Family
AT1G17260	AHA10.1	-3.082	0.003	Belongs to H+-APTase gene family, involved in proanthocyanidin biosynthesis, disturbs the vacuolar biogenesis and acidification process.
AT3G24065	AT3G24065.1	-3.062	0.003	Plant self-incompatibility protein S1 family
AT1G33340	AT1G33340.1	-2.908	0.022	ENTH/ANTH/VHS superfamily protein
AT2G02061	AT2G02061.1	-2.906	0.005	Nucleotide-diphospho-sugar transferase family protein
AT5G02760	AT5G02760.1	-2.896	0.002	Encodes a phosphatase that functions in sustaining proper leaf longevity by suppressing or perturbing SARK-mediated senescence signal transduction.
AT3G17010	AT3G17010.1	-2.845	0.043	transcriptional factor B3 family protein, contains Pfam profile PF02362: B3 DNA binding domain.
AT4G33145	AT4G33145.1	-2.79	0.022	hypothetical protein
AT4G02550	AT4G02550.4	-2.787	0.042	Myb/SANT-like DNA-binding domain protein
AT3G19780	AT3G19780.1	-2.76	0.036	hypothetical protein
AT2G13650	GONST1.1	-2.68	0.016	GOLGI NUCLEOTIDE SUGAR TRANSPORTER 1 (GONST1)
AT1G13710	CYP78A5.1	-2.633	0.014	Encodes the cytochrome P450 CYP78A5 monooxygenase.
AT3G56890	AT3G56890.1	-2.624	0.049	F-box associated ubiquitination effector family protein
AT4G15020	AT4G15020.2	-2.608	0.038	hAT transposon superfamily
AT5G59950	AT5G59950.5	-2.511	0.014	RNA-binding (RRM/RBD/RNP motifs) family protein
AT3G08520	AT3G08520.1	-2.49	0.044	Ribosomal protein L41 family
AT2G14210	AGL44.1	-2.487	0.034	MADS box gene, transcription factor
AT1G68640	PAN.1	-2.481	0.008	Encodes bZIP-transcription factor. PAN is essential for AG activation in early flowers of short-day-grown plants.
AT1G06040	STO.2	-2.441	0.048	Encodes salt tolerance protein (STO) which confers salt tolerance to yeast cells.Sequence has similarities to CONSTANS.
AT4G26680	AT4G26680.2	-2.417	0.007	Tetratricopeptide repeat (TPR)-like superfamily protein
AT1G56020	AT1G56020.1	-2.416	0.017	serine/arginine repetitive matrix-like protein
AT2G41510	CKX1.1	-2.39	0.016	It encodes a protein whose sequence is similar to cytokinin oxidase/dehydrogenase, which catalyzes the degradation of cytokinins.
AT1G02900	RALF1.1	-2.377	0.009	Member of a diversely expressed predicted peptide family showing sequence similarity to tobacco Rapid Alkalinization Factor (RALF)

AT3G01060	AT3G01060.1	-2.362	0.002	lysine-tRNA ligase
AT2G27630	AT2G27630.1	-2.349	0.029	Ubiquitin carboxyl-terminal hydrolase-related protein
AT2G05830	AT2G05830.3	-2.315	0.042	Encodes a 5-methylthioribose-1-phosphate isomerase.
AT2G34150	WAVE1.2	-2.309	0.001	Encodes a member of the SCAR family.These proteins are part of a complex (WAVE) complex.
AT1G79820	SGB1.1	-2.302	0.044	Major facilitator superfamily protein
AT2G42660	AT2G42660.1	-2.236	0.011	Homeodomain-like superfamily protein
AT5G47600	AT5G47600.1	-2.213	0.015	HSP20-like chaperones superfamily protein
AT2G14680	MEE13.3	-2.209	0.016	myosin heavy chain-like protein
AT5G33290	XGD1.1	-2.205	0.021	Acts as a xylogalacturonan xylosyltransferase within the XGA biosynthesis pathway. Involved in pectin biosynthesis.
AT5G12040	AT5G12040.2	-2.204	0.04	Nitrilase/cyanide hydratase and apolipoprotein N-acyltransferase family protein
AT1G70920	HB18.1	-2.174	0.05	homeobox-leucine zipper protein 18
AT3G48180	AT3G48180.1	-2.142	0.006	CDP-diacylglycerol-glycerol-3-phosphate 3-phosphatidyltransferase
AT4G06634	AT4G06634.1	-2.117	0.041	Encodes an ABA responsive C2H2-type zinc finger transcription factor with both transcriptional repression and activation domains.
AT3G10890	AT3G10890.1	-2.115	0.049	Encodes an endo beta mannanase that is localized to the apoplast and involved in glutathione mediated cadmium tolerance.
AT5G65170	AT5G65170.1	-2.092	0.014	VQ motif-containing protein
AT5G58610	AT5G58610.1	-2.073	0.021	PHD finger transcription factor
AT2G41720	EMB2654.1	-2.069	0.009	Encodes a pentatricopeptide repeat protein that is essential for trans-splicing of a chloroplast small ribosomal subunit transcript.
AT4G12450	AT4G12450.1	-2.022	0.028	zinc finger (C2H2 type) family protein
AT5G02040	PRA1.A1.2	-2.022	0.021	prenylated RAB acceptor 1.A1
AT1G07180	NDA1.1	-2.019	0.031	Internal NAD(P)H dehydrogenase in mitochondria.
AT4G23350	AT4G23350.1	-2.018	0.02	transmembrane protein, putative (DUF239)
AT1G65110	AT1G65110.1	-2.015	0.037	Ubiquitin carboxyl-terminal hydrolase-related protein
AT4G16550	AT4G16550.1	-2.008	0.022	HSP20-like chaperone
AT5G16230	AT5G16230.1	-1.995	0.04	Encodes one of two Δ9 palmitoyl-ACP desaturases responsible for the biosynthesis of ω-7 fatty acids in the maturing endosperm.
AT2G34920	EDA18.1	-1.96	0.009	RING/U-box superfamily protein
AT1G76705	AT1G76705.1	-1.955	0.046	calmodulin binding protein
AT2G16660	AT2G16660.1	-1.951	0.045	Major facilitator superfamily protein
AT2G25050	AT2G25050.2	-1.951	0.026	actin-binding FH2 (formin 2) family protein
AT3G13990	AT3G13990.1	-1.94	0.024	dentin sialophosphoprotein, putative (DUF1296)
AT2G33000	AT2G33000.1	-1.932	0	ubiquitin-associated (UBA)/TS-N domain-containing protein-like protein
AT2G03480	QUL2.2	-1.925	0.024	Encodes QUASIMODO2 LIKE2 (QUL2), a paralog of QUASIMODO2 (QUA2).

AT1G33102	AT1G33102.1	-1.892	0.048	hypothetical protein
AT3G48500	PDE312.2	-1.883	0.034	Nucleic acid-binding, OB-fold-like protein
AT3G17950	AT3G17950.2	-1.874	0.046	transmembrane protein
AT2G42950	AT2G42950.1	-1.863	0.047	Magnesium transporter CorA-like family protein
AT5G47650	NUDT2.1	-1.829	0.019	Encodes an ADP-ribose pyrophosphatase that confers enhanced tolerance to oxidative stress.
AT1G18750	AGL65.1	-1.801	0.043	Encodes a member of the MIKC (MADS box, Keratin binding domain, and C terminal domain containing)family of transcriptional regulators.
AT5G43380	TOPP6.2	-1.801	0.042	encodes a type I serine/threonine protein phosphatase expressed in expressed in roots, rosettes and flowers.
AT3G05380	ALY2.3	-1.785	0.028	ALWAYS EARLY 2
AT4G18900	AT4G18900.1	-1.778	0.019	Transducin/WD40 repeat-like superfamily protein
AT5G48490	AT5G48490.1	-1.766	0.049	Encodes a protein with similarity to a lipid transfer protein that may contribute to systemic acquired resistance.
AT4G33740	AT4G33740.3	-1.757	0.044	myb-like protein X
AT3G16175	AT3G16175.1	-1.745	0.014	Thioesterase superfamily protein
AT3G19680	AT3G19680.1	-1.732	0.002	hypothetical protein (DUF1005)
AT3G52050	AT3G52050.5	-1.727	0.02	5-3 exonuclease family protein
AT3G52340	SPP2.2	-1.718	0.047	sucrose-phosphatase (SPP2)
AT3G24020	AT3G24020.1	-1.706	0.007	Disease resistance-responsive (dirigent-like protein) family protein
AT5G67090	AT5G67090.1	-1.7	0.025	Encodes a subtilisin-like serine protease with in vitro protease activity.
AT1G17050	SPS2.1	-1.689	0.024	Encodes one of the two paralogous solanesyl diphosphate synthases that assemble the side-chain of plastoquinone-9 in plastids.
AT1G14350	FLP.2	-1.676	0.007	Encodes a putative MYB transcription factor involved in stomata development
AT4G09820	TT8.1	-1.667	0.042	TT8 is a regulation factor that acts in a concerted action with TT1, PAP1 and TTG1 on the regulation of flavonoid pathways.
AT2G20050	AT2G20050.1	-1.66	0.034	protein phosphatase 2C and cyclic nucleotide-binding/kinase domain-containing protein
AT4G26800	AT4G26800.1	-1.641	0.028	Pentatricopeptide repeat (PPR) superfamily protein
AT1G67920	AT1G67920.1	-1.64	0.028	hypothetical protein
AT5G44740	POLH.2	-1.633	0.01	Y-family DNA polymerase. Catalyses translesion synthesis in response to UV damage. Functionally interacts with PCNA2.
AT5G13660	AT5G13660.2	-1.621	0.024	N-lysine methyltransferase
AT5G47380	AT5G47380.1	-1.611	0.032	electron transporter, putative (Protein of unknown function, DUF547)
AT2G31450	ATNTH1.1	-1.607	0.006	DNA glycosylase superfamily protein
AT1G32520	AT1G32520.1	-1.6	0.017	TLDc domain protein
AT3G06830	AT3G06830.1	-1.599	0.042	Plant invertase/pectin methylesterase inhibitor superfamily
AT5G45840	AT5G45840.1	-1.595	0.036	Encodes a leucine-rich-repeat RLK that is localized to the plasma membrane of pollen tubes.

AT5G66520	AT5G66520.1	-1.59	0.003	Encodes a pentatricopeptide repeat protein involved in chloroplast mRNA editing. Mutants display defects in C-U editing of ndhB.
AT1G14400	UBC1.2	-1.583	0.017	ubiquitin carrier protein
AT3G14940	PPC3.1	-1.563	0.037	Encodes a cytosolic phosphoenolpyruvate carboxylase (PEPC) that has activity when expressed in E.coli.
AT5G61920	AT5G61920.2	-1.562	0.005	FLX-like protein
AT4G08950	EXO.1	-1.55	0.023	Phosphate-responsive 1 family protein
AT1G49630	PREP2.3	-1.542	0.021	Zinc metalloprotease pitrilysin subfamily A. Signal peptide degrading enzyme targeted to mitochondria and chloroplasts.
AT4G16695	AT4G16695.2	-1.541	0.021	transmembrane protein
AT3G15170	CUC1.1	-1.54	0.033	Encodes a transcription factor involved in shoot apical meristem formation and auxin-mediated lateral root formation.
AT1G25250	IDD16.1	-1.537	0.022	Encodes a transcription factor that regulates auxin biosynthesis and transport.
AT1G76065	AT1G76065.1	-1.534	0.02	LYR family of Fe/S cluster biogenesis protein
AT3G08820	AT3G08820.1	-1.516	0.037	Pentatricopeptide repeat (PPR) superfamily protein
AT5G13650	AT5G13650.2	-1.515	0.045	Encodes SVR3, a putative chloroplast TypA translation elongation GTPase.
AT4G36580	AT4G36580.1	-1.514	0.044	AAA-type ATPase family protein
AT1G09170	AT1G09170.1	-1.51	0.003	P-loop nucleoside triphosphate hydrolases superfamily protein with CH (Calponin Homology) domain-containing protein
AT5G47360	AT5G47360.1	-1.508	0.05	Tetratricopeptide repeat (TPR)-like superfamily protein
AT5G23730	AT5G23730.1	-1.507	0.022	Encodes REPRESSOR OF UV-B PHOTOMORPHOGENESIS 2 (RUP2). Functions as a repressor of UV-B signaling.

Supplemental Table 4. Functional categorization of 'Biological Process' GO Terms.

GO Slim Term	Total # GPs	% (all)	% (Figure 3)
GO:0009987 cellular process	191	0.1402	-
GO:0008152 metabolic process	179	0.1314	-
GO:0009058 biosynthetic process	93	0.0683	-
GO:0008150 biological_process *	83	0.0609	-
GO:0006139 nucleobase-containing compound metabolic process	72	0.0529	0.0882
GO:0006950 response to stress	67	0.0492	0.0821
GO:0007275 multicellular organism development	58	0.0426	0.0711
GO:0019538 protein metabolic process	51	0.0374	0.0625
GO:0006810 transport	41	0.0301	0.0502
GO:0016043 cellular component organization	41	0.0301	0.0502
GO:0009628 response to abiotic stimulus	39	0.0286	0.0478
GO:0000003 reproduction	38	0.0279	0.0466
GO:0006464 cellular protein modification process	36	0.0264	0.0441
GO:0009791 post-embryonic development	34	0.0250	0.0417
GO:0009605 response to external stimulus	33	0.0242	0.0404
GO:0009719 response to endogenous stimulus	32	0.0235	0.0392
GO:0007154 cell communication	31	0.0228	0.0380
GO:0007165 signal transduction	26	0.0191	0.0319
GO:0009056 catabolic process	23	0.0169	0.0282
GO:0009607 response to biotic stimulus	21	0.0154	0.0257
GO:0005975 carbohydrate metabolic process	18	0.0132	0.0221
GO:0009653 anatomical structure morphogenesis	17	0.0125	0.0208
GO:0009908 flower development	16	0.0117	0.0196
GO:0030154 cell differentiation	14	0.0103	0.0172
GO:0006629 lipid metabolic process	12	0.0088	0.0147
GO:0040007 growth	12	0.0088	0.0147
GO:0019748 secondary metabolic process	10	0.0073	0.0123
GO:0006412 translation	9	0.0066	0.0110

GO:0006259 DNA metabolic process	8	0.0059	0.0098
GO:0007049 cell cycle	8	0.0059	0.0098
GO:0009790 embryo development	7	0.0051	0.0086
GO:0016049 cell growth	7	0.0051	0.0086
GO:0009991 response to extracellular stimulus	6	0.0044	0.0074
GO:0009856 pollination	5	0.0037	0.0061
GO:0006091 generation of precursor metabolites and energy	4	0.0029	0.0049
GO:0008219 cell death	4	0.0029	0.0049
GO:0015979 photosynthesis	4	0.0029	0.0049
GO:0019725 cellular homeostasis	4	0.0029	0.0049
GO:0009606 tropism	3	0.0022	0.0037
GO:0009838 abscission	2	0.0015	0.0025
GO:0007267 cell-cell signaling	1	0.0007	0.0012
GO:0009875 pollen-pistil interaction	1	0.0007	0.0012
GO:0040029 regulation of gene expression, epigenetic	1	0.0007	0.0012

Table S5: List of oligonucleotides used in this work.

	**Real-time PCR**	
UGT85A3	RT 2478 fw	TGTCAAGAGGGGAGAGGTTG
	RT 2479 rev	TTTATCACCGACGAACCACA
UGT85A3	Atp_6079 fw	CTTCCACGTCACCTTCGTCA
	Atp_6080 rev	TCCGGAACACCAAGCTCTTC
UGT73C1	RT 1500 fw	TGCAGATACTAAAAGCCGGTGTG
	RT 1501 rev	CTTCTTTACTCCTTCTTTATCCACCAG
	**Cloning**	
UGT73C1	AtP_4903 fw	GGGGACAAGTTTGTACAAAAAAGCAGGCTTC ATGGCATCGGAATTTCGTCCT
	AtP_4904 rev	GGGGACCACTTTGTACAAGAAAGCTGGGTC TCATTTCTTGGGTTGTTCTAATTGCA
UGT85A3	AtP_4907 fw	GGGGACAAGTTTGTACAAAAAAGCAGGCTTC ATGGGATCCCGTTTTGTTTCT
	AtP_4908 rev	GGGGACCACTTTGTACAAGAAAGCTGGGTC TTACGTGTTAGGGATCTTTCCCA
	**Genotyping**	
UGT85A3 WT allele	AtP_6079 fw	CTTCCACGTCACCTTCGTCA
	AtP_6125 rev	CCCAAGCACTCAGTCTCCTC
ugt85a3-1 mutant allele	AtP_5960 sail LB	TAGCATCTGAATTTCATAACCAATCTCGATACAC
	AtP_6125 rev	CCCAAGCACTCAGTCTCCTC
ugt85a3-2 mutant allele	AtP_4100 on T-DNA	GGCCGCGGGAATTCGATT
	AtP_4913 rev	tcaatccattttactaatccagggt
UGT73C1 WT allele	AtP_4903 fw	GGGGACAAGTTTGTACAAAAAAGCAGGCTTC ATGGCATCGGAATTTCGTCCT
	AtP_4904 rev	GGGGACCACTTTGTACAAGAAAGCTGGGTC TCATTTCTTGGGTTGTTCTAATTGCA
ugt73c1 mutant allele	AtP_4909 fw	AGAGAGTTCCTCATGGCATCG
	AtP_4290 salk LB	ATTTTGCCGATTTCGGAAC
